# Impact of COVID-19 on obstetric anesthesia: a systematic review

**DOI:** 10.1186/s42077-021-00188-w

**Published:** 2021-11-02

**Authors:** Shrief Nasr

**Affiliations:** 1 Department of Anesthesia, Queen Elizabeth Hospital - King’s Lynn, Norfolk, UK; 2 Department of Anesthesia, Intensive Care and Pain Management, Faculty of Medicine, Ain Shams University, Cairo, Egypt

**Keywords:** COVID-19, SARS-CoV-II, Obstetric anesthesia, Pregnancy, Practice recommendations

## Abstract

With an increase in Coronavirus Disease 2019 (Covid-19) incidents around the world, it has become more important than ever to be prepared for the uncertain context of labor and delivery in obstetrics. As medical staff did not encounter such a situation previously, no prior knowledge and guidelines were present to assist them. During the care of obstetric women infected with COVID-19 as well as those who are suspected of COVID-19 infection, there are two objectives, the care of asymptomatic to severely sick pregnant and postpartum women and preventing exposure of medical professionals and others during childbirth hospitalization. The focus of this review is to provide anesthesiologists who are dealing with infected pregnant mothers with some facts or, as data is insufficient, expert opinion, with an emphasis on awareness and optimal medical obstetric anesthesia training. This review will provide possible recommendations for the obstetric anesthesiologists when treating infected obstetric women and these recommendations also help anesthesia providers to prepare themselves for future pandemics.

## Background

In December 2019, a virus known as SARS-CoV-II has emerged from Wuhan and rapidly spread to other countries. The infection caused by SRAS-COV-II is termed COVID-19. The common symptoms of this infection are flu, fever, diarrhea, fatigue, and cough (Huang et al. 2020; Chan and Brownstein 2020). COVID-19 caused 100 thousand fatalities around the world due to its high transmissible nature and was termed a pandemic (Melnick and Ioannidis 2020). Though elective procedures could be delayed preventing exposure and save personal protective equipment (PPE) during the pandemic (Critical care and anaesthesia service reorganisation 2020). All the other medical treatments were postponed during lockdown situations but the obstetric activity could not be delayed or postponed. The susceptibility of pregnant women for developing COVID-19 infection is more due to physiological changes in their immune and cardiovascular systems (Breslin et al. 2020). Thus, special care is required for pregnant women before, during, and after delivery or C-section. Before this pandemic, obstetric anesthesiologists were only encountered with healthy pregnant mothers and focused to provide best practice standards and choices for what patients want for a safe and better birth experience, although they encountered sick pregnant women sometimes but sick in terms of maternal morbidity related to obesity, aging, eclampsia, cardiovascular diseases, and postpartum hemorrhage (PPH) (Mhyre et al. 2011; Jain and Shirodkar 2019). These conditions are already appearing as a threat to modern society; now, the emergence of COVID-19 worsens the situation. Anesthesiologists have to deal with infected pregnant women, when there are no prior studies about the disease severity in pregnant women or guidelines are present to adopt for preventing the spread of infection from affected mother to hospital staff and neonatal. Moreover, from the previous 1.5 years, it has been observed that an enormous number of people are infected with SARS-CoV-II that increases the demand of hospitals, intensive care units (ICUs), and hospital staff including nurses and anesthesiologists. Moreover, obstetric anesthetists did not face this situation previously. They are not trained to deal with infected mothers. So, some precautionary measures should be adopted by anesthetists (Yamakage 2021). In this study, we will briefly discuss how anesthetists should prepare themselves in the context of such a pandemic.

## Clinical features of COVID-19 in pregnant women

Pregnant women show similar symptoms of COVID-19 as observed on non-pregnant healthy women. However, most of these characteristic symptoms could be related to pregnancy and labor symptoms (Chen et al. 2020a). Myalgias and diarrhea are common indications of latent labor; moreover, pre-eclampsia could induce severe pain in the head; difficulty in breathing is common throughout pregnancy and labor. Moreover, tachycardia and fever could be caused by chorioamnionitis. All these signs lead doctors to neglect COVID-19 infection as a probable disease. Furthermore, COVID-19-infected women may be asymptomatic until they are admitted to the hospital for labor and delivery. This situation poses a considerable risk of transmission of the virus to the patient’s family (the infant) and any healthcare workers engaged in their care (Breslin et al. 2020; Wastnedge et al. 2021).

## Selection of articles

**Fig. 1:**
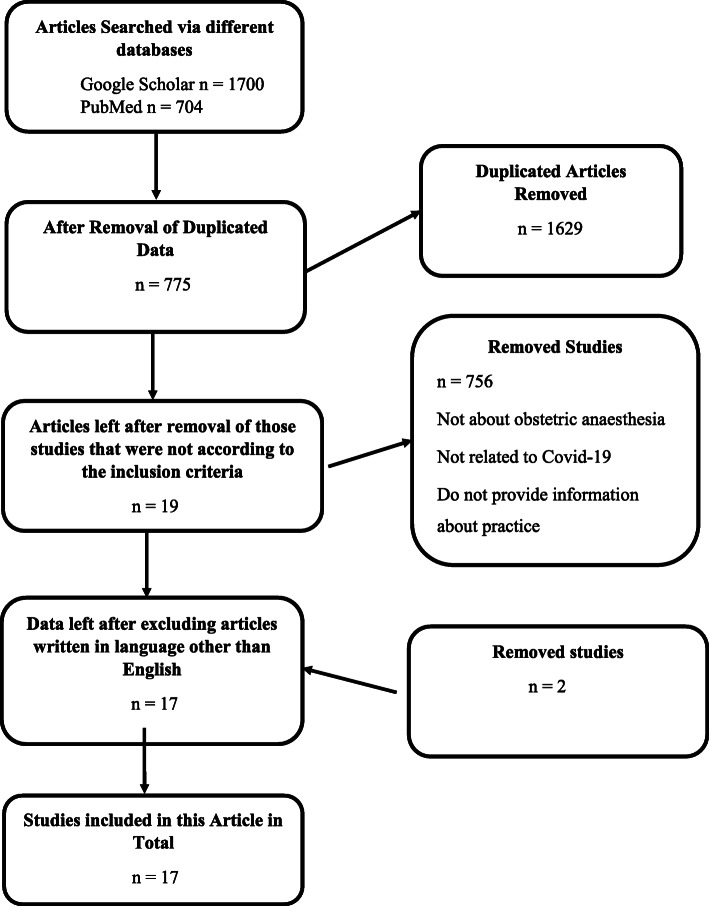
Summary of the searched information and the method selected for data collection

Relevant articles for this systematic review were searched from two databases including Google Scholar and PubMed. Studies only from peer-reviewed journals were selected. Different keywords including obstetric anesthesia, Covid-19, obstetric anesthesia AND Covid-19, and recommendations for anesthesiologists in Covid-19 were used to search relevant data. Some data on the current related recommendations and guidance from the relevant medical societies’ websites including the Royal College of Obstetricians and Gynaecologists (RCOG), Society for Obstetric Anesthesia and Perinatology (SOAP), and the American Society of Anesthesiologists (ASA) were also reviewed. The inclusion criteria were set that only peer-reviewed articles about practice recommendations and consideration for obstetric anesthesia in the pandemic of COVID-19 were included in this study. Figure 1 shows the flowchart diagram of the number of articles obtained from different databases and selected for this review.

## Results

The following are some recommendations, guidelines, and considerations obtained from studies included in this review. Table 1 represents the studies included in the “Results” section with their key findings.

**Table 1:** List of articles and their key findings included in this study

Sr. no.	Title	Findings	Reference
1	What obstetricians should know about obstetric anesthesia during the COVID-19 pandemic	Avoid general anesthesia, opt neuraxial analgesia, avoid emergent C-section childbirth	(Ring et al. 2020)
2	Neuraxial procedures in COVID-19 positive parturients: a review of current reports	The neuraxial procedure should adopt for COVID-19 infected pregnant women	(Bauer et al. 2020a)
3	Safety and efficacy of different anesthetic regimens for parturients with COVID-19 undergoing Cesarean delivery: a case series of 17 patients	Epidural and general anesthesia is safe for C-section delivery, PPE required for medical staff safety	(Chen et al. 2020b)
4	Anesthetic Management for Emergent Cesarean Delivery in a Parturient with Recent Diagnosis of Coronavirus Disease 2019 (COVID-19): A Case Report	Combined spinal and epidural anesthesia is effective and ensures the safety of the patient	(Song et al. 2020)
5	Spinal anesthesia for patients with Coronavirus Disease 2019 and possible transmission rates in anesthetists: retrospective, single-center, observational cohort study	Administration of spinal anesthesia was safe for COVID-19-infected patients and BSL-3 PPE are important for obstetric anesthesiologists	(Zhong et al. 2020)
6	Obstetric anesthesia care and Covid-19	Neuraxial anesthesia recommended	(Kader and Siddik-Sayyid 2020)
7	Practice recommendations on neuraxial anesthesia and peripheral nerve blocks during the COVID-19 pandemic	Anesthesia care should provide only for urgent and critical cases	(Uppal et al. 2020)
8	Safe anesthesia and analgesia for obstetric patients in COVID 19 pandemic	Special facilities are required for the care of COVID-19 pregnant women such as video laryngoscope should be preferred over direct laryngoscope to avoid the direct contact of medical staff with infected women	(Ismail and Aman 2020)
9	Putting It All Together: Clinical Considerations in the Care of Critically Ill Obstetric Patients with COVID-19		(Oxford-Horrey et al. 2020)
10	Successful Anesthetic Management in Cesarean Section for Pregnant Woman with COVID-19	Spinal anesthesia should prefer over general anesthesia in case of a C-section	(Hani et al. 2020)
11	Considerations and strategies in the organisation of obstetric anesthesia care during the 2019 COVID-19 outbreak in Singapore	Communication is necessary between staff, institutional leadership, and paramedics to get more and more knowledge and practices against COVID-19	(Lee et al. 2020)
12	Anesthetic Management of Pregnant Patients With COVID-19	No vertical transmission of COVID-19 infection from mother to infant is possible, epidural anesthesia should be considered and continuous monitoring of platelet count to prevent the risk of thrombocytopenia	(Daykin and Moore 2020)
13	Considerations and Recommendations for Obstetric Anesthesia Care During COVID-19 Pandemic-Saudi Anesthesia Society Guidelines	Special care and facilities are required for the care of COVID-19 pregnant women	(Alyamani et al. 2020)
14	COVID-19: Obstetric anesthesia care considerations	Patients should inform hospital staff if infected prior to hospital admission, use of isolation rooms and PPEs	(Herman et al. 2021)
15	What obstetricians should know about obstetric anesthesia during the COVID-19 pandemic	Do not opt for general anesthesia, prefer early neuraxial analgesia Use of PPEs	(Ring et al. 2020)
16	Obstetric Anesthesia During the Coronavirus Disease 2019 Pandemic		(Bauer et al. 2020b)
17	Novel coronavirus SARS-CoV-2 and COVID-19. Practice recommendations for obstetric anesthesia: what we have learned thus far		(Bampoe et al. 2020)

## Recommendations and considerations

### Personal protective equipment

The treatment or dealing with COVID-19 positive or suspected patients required PPE. The type of PPE to use will be determined by the mode of transmission risk (Cook 2020). The mode of transmission of COVID-19 infection-causing virus is via respiratory droplets and interaction with contaminated substances and surfaces. After that, replication of the virus occurs in the airway epithelium of the lungs. Though SARS-CoV-II is not considered to be an airborne virus, it could transmit through the air in some conditions or clinical settings where aerosols are produced. Airborne precautions are recommended for aerosol-generating procedures (AGPs). Exhaling deeply during labor and pushing during delivery does not generate aerosols (Daykin and Moore 2020).

### Management of obstetric women infected with COVID-19

The Royal College of Obstetricians and Gynaecologists (RCOG) has provided detailed guidelines for the treatment of COVID-19-positive obstetric patients (Daykin and Moore 2020; Coronavirus (COVID-19) infection in pregnancy 2020). The following are some general management practices.

I. The personnel of the maternity ward should be informed when a COVID-19 infected, or suspected woman is admitted. The members of the maternity ward are senior obstetricians, senior anesthesia practitioners, midwife-in-charge, and senior neonatologists or pediatricians.
II. Temperature, respiration rate, and oxygen saturation should be monitored after every hour. Oxygen saturation must be of more than 94% and optimization of oxygen therapy as needed.
III. When it comes to intravenous fluid treatment, be cautious. Monitoring of fluid status of women with mild-severe infection symptoms must be done carefully through input-output chart after every hour. This will help to find a link between COVID-19 and acute respiratory distress syndrome. Fluid boluses in the range of 250–500 mL should be administered with re-evaluation instead of continuing with fluid restoration.
IV. As much as possible, only few staff members should visit the patient room and any possible direct interaction with the patient must be kept to a minimum, and hospitals should establish special plans for workers in case of an emergency.
V. Continual cardiotocography monitoring should be used to monitor the fetus.
VI. COVID-19 increased the possibility of thromboembolism. Below are the measures that should be adopted for all COVID-19 infected pregnant women admitted in hospitals:
  - If childbirth is expected within 12 hours, prophylactic low-molecular-weight heparin should be given. When making this decision, the incidence of neuraxial analgesia or general anesthesia, delivery, or possible problems must be taken into consideration, as well as a specific risk-benefit assessment should be performed.
  - After hospital discharge, all COVID-19-positive pregnant women should get thromboprophylaxis for 10 days. Suggest a longer course of thromboprophylaxis for women with continual morbidity.
  - If COVID-19-positive women are admitted to the hospital before the completion of 6 weeks of postpartum, it is advised to administer them with thromboprophylaxis for the duration of their stay and also 10 days after their discharge from the hospital. For women with considerable persistent infection, consider prolonging this to 6 weeks postpartum.
VII. Anxiety and postnatal depression are already more common in pregnant and postpartum women. The situation of COVID-19 may be harmful to maternal and fetal health, exacerbating mental health symptoms. Where visitor limitations are in place, women may feel more alone and require greater mental counseling.

The Society for Obstetric Anesthesia and Perinatology (SOAP) and the American Society of Anesthesiologists (ASA) have also issued recommendations and guidelines for obstetric anesthesia in COVID-19 (Katz et al. 2021; Zhao et al. 2020; Perinatology 2020). The following recommendations are summarized according to the guidelines provided by these societies.

#### Labor analgesia

There are limited published resources to support practical recommendations about the use of labor analgesia. The available data are usually based on the transmission risks associated with SARS-CoV-II and related viruses. These resources are also being combined with the data arising from maternity units caring for COVID-19 patients (Bampoe et al. 2020).

#### Nitrous oxide

The Entonox breathing systems do not require an AGP. Hence, PPE for AGPs is not necessary for the personnel involved in the care of women with either confirmed or suspected cases of COVID-19 and those who want to apply nitrous oxide analgesia in labor. However, there is a risk of infecting the respiratory airway, and therefore, the use of antiviral filters is necessary (Bampoe et al. 2020).

#### Remifentanil

There are no sufficient data of remifentanil as control analgesia in patients suffering from obesity and COVID-19. However, it should be carefully used in labor due to the possibility of developing respiratory depression specifically, for women who show respiratory problems. It should be avoided whose oxygen saturation is less than 95% or it can cause further complications (Herman et al. 2021; Bampoe et al. 2020).

#### Neuraxial analgesia

No data is present regarding the fact that spinal anesthesia and epidural anesthesia should not be used in COVID-19 patients. However, epidural analgesia is preferred for both the COVID-19-confirmed and COVID-19-suspected mothers undergoing labor. On the plus side, epidural analgesia in the labor women with coronavirus provides an added benefit, as to avoid the conversion to general anesthesia, which is an AGP, there is an edge to shift the epidural analgesia to surgical analgesia (Ring et al. 2020; Bauer et al. 2020b).

Some of the initial studies suggested that thrombocytopenia and COVID-19 are correlated because almost one-third of the COVID-19 patients also have thrombocytopenia. Moreover, the intensity of the platelet reduction is directly linked with the severity of the COVID-19 infection in patients. It is recommended that while dealing with the COVID-19 positive patients always look for the platelet count first before opting for the epidural or spinal analgesia (Bampoe et al. 2020).

Regular evaluation of the patient to find out the problems at their early stages is always preferred in case of a labor epidural. Always have an option of the initial resite as there are chances that during the operative events, epidural analgesia may be insufficient to be shifted to the anesthesia (Bampoe et al. 2020).

#### Emergency C-section

While having a COVID-19 positive woman who needs a cesarean section at the emergency, the most critical part for the anesthesia provider is to opt for the PPE and more specifically choosing between the precautionary measures of airborne as well as the respiratory droplets. Airborne precautions are preferred in the case of general anesthesia while droplet precautions are preferred in the case of neuraxial anesthesia. Though, there are possibilities of the conversion to general anesthesia from neuraxial anesthesia during the operation so it is recommended; so, using airborne precautions is always preferred even in the case of neuraxial anesthesia (Bampoe et al. 2020). In order to minimize the risk of getting contaminated during the conversion process to general anesthesia, the clinical professionals should wear N95 or FFP2/3 face masks rather than the regular ones. Clinicians must look for alternative options to opt for in order to avoid the shifting from neuraxial anesthesia to general anesthesia. A speedy spinal anesthetic is always preferred over general anesthesia even during category 1 C-section operations because of its hectic steps as well as the risk of aerosolization in COVID-19 patients. Even in emergency cases, the most skilled anesthesia provider should perform spinal anesthesia to avoid the multiple failed attempts as well as the need of shifting it to general anesthesia (Hani et al. 2020).

#### General anesthesia

As a standard treatment, rapid-sequence induction is recommended to be executed on pregnant ladies. As the air-borne precautionary measure, PPEs are recommended to wear and both the intubation and extubations are included in the AGPs (Bauer et al. 2020a; Lee et al. 2020).

1. The usage of cannulas with a high-flow rate for the purpose of either pre-oxygenation as well as apnoeic oxygenation is not recommended.
2. Video laryngoscopy should always be preferred over direct laryngoscopy. It should always be given as the first-line treatment along with the intubation by the most skilled anesthesia provider. Its main advantage is that it helps in maintaining a safe distance from the airway passage of the patient. Also, only upon the inflation of the endotracheal tube cuff do the ventilation of the patient (Ismail and Aman 2020; Oxford-Horrey et al. 2020).
3. Rapid desaturation is always expected because of the affected respiratory system and its functions.
4. In order to prevent the contamination caused by respiratory secretions, always wear two pairs of gloves and remove one right after securing the endotracheal tube cuff (Zhao et al. 2020).
5. The use of the auscultation technique for the confirmation of the endotracheal tube is not recommended. Instead, the use of other techniques like end-tidal CO_2_ and chest rising technique is recommended for this purpose (Ismail and Aman 2020).
6. As extubation is AGP, it is always preferred to limit the number of individuals in the patient’s room at the time of extubation.
7. Always be critical in opting for the post-operative site for the mother according to the clinical situation of the mother. Discussion with the team of the intensive care unit is recommended for this purpose (Ismail and Aman 2020; Perinatology 2020).

#### Postoperative and postnatal care

1. Patients/individuals who are awake and are suspected of COVID-19 or positive for COVID-19 are asked to wear fluid-protective facemasks.
2. Although nonsteroidal anti-inflammatory drugs (NSAIDs) can negatively impact the condition of the patients having SARS-CoV-II but still there is not any experimental evidence to this statement. The present guideline regarding this is that patients can use NSAIDs for the treatment of postoperative analgesia (Zhong et al. 2020).
3. Both the patients in healthcare organizations and the healthcare staff should be provided with extra support throughout the pandemic situation. As postnatal women as well as pregnant women are at enhanced risks of having severe depression, the mental health evaluation of both the staff and the patients should be done from time to time (Critical care and anaesthesia service reorganisation 2020; Zhao et al. 2020).

#### Breastfeeding

At present, there are not many studies regarding the safety measure of breastfeeding and also that either there is no requirement to separate the baby and mother due to safety measures. As it is a known fact that the route of transmission for viral particles in the respiratory droplets and not the breastmilk so the only concern here is that the mother is required to maintain her own cleanliness and also wear a protective mask to protect their children. The present guideline regarding newborns from either the suspected or SARS-CoV-II-positive mothers is that the mother and the newborn baby should not be separated (Daykin and Moore 2020; Bauer et al. 2020b).

## Broader considerations

As nowadays the major emphasis of healthcare institutes is on the COVID-19, it does not mean that the already prevailing healthcare standards in other sectors should be neglected. In 2016, there was an Ebola epidemic in Sierra Leone, and during that epidemic, it was estimated that because of this outbreak, a notable reduction in postnatal and antenatal care in women at the healthcare centers was seen. This reduction further resulted in a 34% increased mortality rate associated with facility-related maternal deaths and a 24% rise in stillbirth frequency (Yerger et al. 2020). There is a dire need for healthcare professionals to ensure the fact that even during pandemic situations, fetal as well as maternal outcomes should be unaffected.

## Conclusions

The COVID-19 poses serious threats to the anesthesia provider and other medical staff when they encounter COVID-19 positive or under investigation pregnant women in hospitals. There is a need to formulate special care guidelines and steps to prepare anesthesiologists. Special plans and common guidelines should be formulated to train medical staff of maternity wards for such kinds of scenarios.

The traditional parturient cycle has been interrupted, and now, the medical staff has to transfer puerperal or post-cesarean patients to units that have never encountered such patients before. The list of new obstacles for patients may go on for dozens of pages, but it is unclear whether or not the quality of care is in jeopardy. In a nutshell, we must learn to work in low-resource environments in some ways. Various recommendations and publications on the safety of neuraxial surgeries in pregnant COVID-19 women have been published to guide medical staff. What if a COVID-19 positive woman develops a complication, like postpartum hemorrhage, or if a non-COVID-19 woman requires ICU admission? Are we currently capable and equipped to provide a high level of care? Returning to the highest levels of care for obstetric patients is really the challenge yet.

## Data Availability

All the peer-reviewed publications and methods are mentioned with references.

## Abbreviations

BSL-3: Biosafety level 3
COVID-19: Coronavirus disease 2019
SARS CoV-II: Severe acute respiratory syndrome coronavirus-II
PPE: Personal protective equipment
PPH: Postpartum hemorrhage
ICUs: Intensive care units
SOAP: Society for Obstetric Anesthesia and Perinatology
RCOG: Royal College of Obstetricians and Gynaecologists
ASA: American Society of Anesthesiologists
AGPs: Aerosol-generating procedures
NSAIDs: Nonsteroidal anti-inflammatory drugs
